# The Cost and Impact of Scaling Up Pre-exposure Prophylaxis for HIV Prevention: A Systematic Review of Cost-Effectiveness Modelling Studies

**DOI:** 10.1371/journal.pmed.1001401

**Published:** 2013-03-12

**Authors:** Gabriela B. Gomez, Annick Borquez, Kelsey K. Case, Ana Wheelock, Anna Vassall, Catherine Hankins

**Affiliations:** 1 Department of Global Health, Academic Medical Centre, University of Amsterdam and Amsterdam Institute for Global Health and Development, The Netherlands; 2 School of Public Health, Imperial College London, United Kingdom; 3 Centre for Patient Safety and Service Quality, Imperial College London, United Kingdom; 4 London School of Hygiene and Tropical Medicine, United Kingdom; Harvard School of Public Health, United States of America

## Abstract

**Background:**

Cost-effectiveness studies inform resource allocation, strategy, and policy development. However, due to their complexity, dependence on assumptions made, and inherent uncertainty, synthesising, and generalising the results can be difficult. We assess cost-effectiveness models evaluating expected health gains and costs of HIV pre-exposure prophylaxis (PrEP) interventions.

**Methods and Findings:**

We conducted a systematic review comparing epidemiological and economic assumptions of cost-effectiveness studies using various modelling approaches. The following databases were searched (until January 2013): PubMed/Medline, ISI Web of Knowledge, Centre for Reviews and Dissemination databases, EconLIT, and region-specific databases. We included modelling studies reporting both cost and expected impact of a PrEP roll-out. We explored five issues: prioritisation strategies, adherence, behaviour change, toxicity, and resistance. Of 961 studies retrieved, 13 were included. Studies modelled populations (heterosexual couples, men who have sex with men, people who inject drugs) in generalised and concentrated epidemics from Southern Africa (including South Africa), Ukraine, USA, and Peru. PrEP was found to have the potential to be a cost-effective addition to HIV prevention programmes in specific settings. The extent of the impact of PrEP depended upon assumptions made concerning cost, epidemic context, programme coverage, prioritisation strategies, and individual-level adherence. Delivery of PrEP to key populations at highest risk of HIV exposure appears the most cost-effective strategy. Limitations of this review include the partial geographical coverage, our inability to perform a meta-analysis, and the paucity of information available exploring trade-offs between early treatment and PrEP.

**Conclusions:**

Our review identifies the main considerations to address in assessing cost-effectiveness analyses of a PrEP intervention—cost, epidemic context, individual adherence level, PrEP programme coverage, and prioritisation strategy. Cost-effectiveness studies indicating where resources can be applied for greatest impact are essential to guide resource allocation decisions; however, the results of such analyses must be considered within the context of the underlying assumptions made.

*Please see later in the article for the Editors' Summary*

## Abstract 2

Gabriela Gomez and colleagues systematically review cost-effectiveness modeling studies of pre-exposure prophylaxis (PrEP) for preventing HIV transmission and identify the main considerations to address when considering the introduction of PrEP to HIV prevention programs.

## Editors' Summary

**Background:**

Every year approximately 2.5 million people are infected with HIV, the virus that causes AIDS. Behavioral strategies like condom use and reduction of sexual partners have been the hallmarks of HIV prevention efforts. However, biological prevention measures have also recently been shown to be effective. These include male circumcision, treatment as prevention (treating HIV-infected people with antiretroviral drugs to reduce transmission), and pre-exposure prophylaxis (PrEP), where people not infected with HIV take antiretroviral drugs to reduce the probability of transmission. Strategies such as PrEP may be viable prevention measure for couples in long-term relationships where one partner is HIV-positive and the other is HIV-negative (HIV serodiscordant couples) or groups at higher risk of HIV infection, such as men who have sex with men, and injection drug users.

**Why Was This Study Done?:**

The findings from recent clinical trials that demonstrate PrEP can reduce HIV transmission have led to important policy discussions and in the US, Southern Africa, and the UK new clinical guidelines have been developed on the use of PrEP for the prevention of HIV infection. For those countries that are considering whether to introduce PrEP into HIV prevention programs, national policy and decision makers need to determine potential costs and health outcomes. Cost-effectiveness models—mathematical models that simulate cost and health effects of different interventions—can help inform such decisions. However, the cost-effectiveness estimates that could provide guidance for PrEP programs are dependent on, and limited by, the assumptions included in the models, which can make their findings difficult to generalize. A systematic comparison of published cost-effectiveness models of HIV PrEP interventions would be useful for policy makers who are considering introducing PrEP intervention programs.

**What Did the Researchers Do and Find?:**

The researchers performed a systematic review to identify published cost-effectiveness models that evaluated the health gains and costs of HIV PrEP interventions. Systematic reviews attempt to identify, appraise, and synthesize all the empirical evidence that meets pre-specified eligibility criteria to answer a given research question by using explicit methods aimed at minimizing bias. By searching databases the authors identified 13 published studies that evaluated the impact of PrEP in different populations (heterosexual couples, men who have sex with men, and injection drug users) in different geographic settings, which included Southern Africa, Ukraine, US, and Peru.

The authors identified seven studies that assessed the introduction of PrEP into generalized HIV epidemics in Southern Africa. These studies suggest that PrEP may be a cost effective intervention to prevent heterosexual transmission. However, the authors note that funding PrEP while other cost-effective HIV prevention methods are underfunded in this setting may have high opportunity costs. The authors identified five studies where PrEP was introduced for concentrated epidemics among men who have sex with men (four studies in the US and one in Peru). These studies suggest that PrEP may have a substantial impact on the HIV epidemic but may not be affordable at current drug prices. The authors also identified a single study that modeled the introduction of PrEP for people who inject drugs in the Ukraine, which found PrEP not to be cost effective.

In all settings the price of antiretroviral drugs was found to be a limiting factor in terms of affordability of PrEP programs. Behavioral changes and adherence to PrEP were estimated to have potentially significant impacts on program effectiveness but the emergence of drug resistance or PrEP-related toxicity did not significantly affect cost-effectiveness estimates. Several PrEP prioritization strategies were explored in included studies and delivering PrEP to populations at highest risk of HIV exposure was shown to improve cost-effectiveness estimates. However, the extra costs of identifying and engaging with high-risk populations were not taken into consideration. The authors note that the geographic coverage of identified studies was limited and that the findings are very dependent on the setting which limits generalizability.

**What Do these Findings Mean?:**

These findings suggest that PrEP could be a cost-effective tool to reduce new HIV infections in some settings. However, the cost-effectiveness of PrEP is dependent upon cost, the epidemic context, program coverage and prioritization strategies, participants' adherence to the drug regimen, and PrEP efficacy estimates. These findings will aid decision makers quantify and compare the reductions in HIV incidence that could be achieved by implementing a PrEP program.

**Additional Information:**

Please access these Web sites via the online version of this summary at http://dx.doi.org/10.1371/journal.pmed.1001401.

- The US National Institute of Allergy and Infectious Diseases has information on HIV/AIDS
- aidsmap provides basic information about HIV/AIDS, summaries of recent research findings on HIV care and treatment, and has a section on PrEP
- Information is available from Avert, an international AIDS charity, on many aspects of HIV/AIDS, including HIV prevention
- AVAC Global Advocacy for HIV Prevention provides information on HIV prevention, including PrEP
- The US Centers for Disease Control and Prevention also has information on PrEP
- The World Health Organization has a page on its WHO-CHOICE criteria for cost-effectiveness

## Introduction

Since the announcement of the results of HIV pre-exposure prophylaxis (PrEP) trials and the HPTN052 early treatment for prevention trial, there have been crucial policy discussions about the use of antiretroviral (ARV) drugs to prevent HIV acquisition or transmission. With regards to PrEP, encouraging results were first reported for men and transgender women who have sex with men in the iPrEX trial [1], which showed a 44% (95% CI 15–63) reduction in HIV acquisition with a daily dose of tenofovir/emtricitabine (TDF/FTC). In two large trials, the Partners PrEP [2] and TDF2 [3] studies, PrEP was found to be effective in reducing the risk of heterosexual HIV transmission using either TDF or TDF/FTC daily (Partners PrEP) and TDF/FTC daily (TDF2). However, FEM-PrEP [4], a trial recruiting heterosexual women in South Africa, Tanzania, and Kenya for daily TDF/FTC was closed prematurely in 2011 for futility as was the oral TDF arm of the VOICE trial [5] in women in South Africa, Uganda, and Zimbabwe. Two topical PrEP trials have tested the efficacy of 1% TDF gel and a third, FACTS001 [6], is currently recruiting women in South Africa. The CAPRISA 004 trial [7] in Kwa Zulu-Natal found that pre- and post-coital vaginal TDF gel reduced women's acquisition risk by 39% (95% CI 6–60) but the VOICE trial stopped its gel arm when it became evident that daily gel use was safe but not effective [8].

Clinical guidance on oral PrEP has already been offered by the US Centers for Disease Control and Prevention, the Southern African HIV Clinicians Society, World Health Organization (WHO), and the British Association for Sexual Health and HIV [9]–[13]. An advisory panel to the US Food and Drug Administration recently recommended oral TDF/FTC for preventive use among people at higher risk of HIV exposure [14]. As PrEP emerges as an option for inclusion in the HIV prevention toolbox, it is important for national policy and decision makers to identify where PrEP may fit best within already established HIV prevention programming (and budgets) and the potential implications of introducing such policy changes. In particular, decision makers need information translating the trial results into potential population-level impact and cost-effectiveness to ensure that any additional investment will have the maximum possible effect on the epidemic.

Economic and mathematical models provide a framework to integrate information on efficacy, effectiveness, costs, and patient outcomes to support decision making and resource allocation [15]. However, due to their complexity, dependence on assumptions made, and inherent uncertainties, generalising results from these models can be difficult. In this review, we aim to assess published cost-effectiveness models that have evaluated the expected health gains and costs of PrEP interventions. Specifically, our objectives are: (1) to describe modelling approaches of cost-effectiveness analyses of PrEP; (2) to compare the effects of epidemiological and cost assumptions on cost-effectiveness results; and (3) to explore the potential impact on cost-effectiveness estimates of five issues raised by policy makers [16]–[18] when considering PrEP implementation: prioritisation, adherence, behaviour change, toxicity, and resistance.

## Methods

We performed a systematic review of the published literature following the protocol available in Text S2 and adhering to the PRISMA guidelines for reporting of systematic reviews (Text S1: PRISMA checklist) [19] and guidelines for appraisal of economic evaluations [20].

### Search Strategy, Inclusion Criteria, and Study Selection

A broad strategy using both MeSH headings and free text, with no language limitations, was used to search PubMed/Medline, ISI Web of Knowledge (including Web of Science, Current Contents Connect, Derwent Innovations Index, CABI: CAB Abstracts, and Journal Citation Reports), Centre for Reviews and Dissemination databases (including DARE - Database of Abstracts of Reviews of Effects, NHS EED - NHS Economic Evaluation Database, and HTA database - health technology assessments), EconLIT, and region-specific databases (African Index Medicus, Eastern Mediterranean Literature (WHO), Index Medicus for South-East Asia Region, LILACS for Latin America). Our searches covered all published research up to the last search performed 14 January 2013 with no limitations on publication date. The following keywords were used: “cost” AND “tenofovir OR pre-exposure prophylaxis OR chemoprophylaxis OR PrEP” AND “HIV.” Citations and bibliographies of full text reports retrieved were reviewed for additional relevant articles. Abstracts from international conferences identified in the searches were also reviewed, as was the website of the International AIDS Economic Network. Experts were consulted for additional studies. We included all modelling studies reporting both cost and impact of a potential roll-out of a PrEP programme. We excluded those studies where costs were not assessed. No restrictions were made on the type of model, geography, mode of transmission, or impact (effectiveness) metric chosen. We included studies looking at both topical and systemic PrEP products. Full published papers were eligible, as well as abstracts from conferences providing sufficient information. Two authors (GBG and AB) screened titles and abstracts to identify potentially relevant articles. Full text reports of these articles were assessed independently for inclusion.

### Data Extraction and Analysis

Data were extracted from selected studies by one reviewer (GBG) into prepared data sheets and independently cross-checked by a second assessor (AB). For conference abstracts selected for inclusion, we contacted the first author listed for further information. Extracted information on the study design included the type of study, viewpoint of analysis, timeframe, setting and population, background HIV prevalence or incidence, mode of HIV transmission, and a detailed description of alternative programmes compared in the studies (baseline scenario and PrEP scenario). We also tabulated data on the impact including risk heterogeneity, efficacy or effectiveness of PrEP, adherence (to programme or individual), behavioural change expected after introduction of PrEP, resistance, toxicity due to PrEP use, and disability-adjusted life year (DALY)/quality-adjusted life year (QALY) assumptions. A description of economic assumptions includes expected drug cost, other service costs, costs above service level, downstream antiretroviral treatment (ART) costs averted, discount rates, and, finally, cost-effectiveness results by metric and the conclusions presented in each publication. Prioritised scenarios were defined as those scenarios where PrEP was offered to specific sub-populations within the population modelled. While providing a critical assessment and narrative review of the studies included, we did not attempt to perform a meta-analysis due to the variability across the studies in reporting outcomes. Therefore, we adjusted estimates of cost-effectiveness for inflation to US$2012 to be able to compare studies from different years [21]. For those studies reporting cost/DALY averted, cost/QALY averted, or cost/life-year saved (LYS), we compared the estimates to a benchmark for cost-effectiveness [22] of one times the gross domestic product per capita (GDP/capita) per DALY averted, per QALY gained, or per LYS, depending on the unit of outcome used by each study. While DALYs, QALYs, and LYS are not equivalent, and decision rules vary by setting, this gives a broad indication of potential cost-effectiveness. The values for current GDP/capita were sourced from the World Bank databank for each country [23]. There is much controversy around decision rules [24], and while the comparison against GDP is the conventional approach, it should be noted that this may not represent the true opportunity cost in countries where less cost-effective health interventions are not being implemented at scale.

## Results

**Figure 1:**
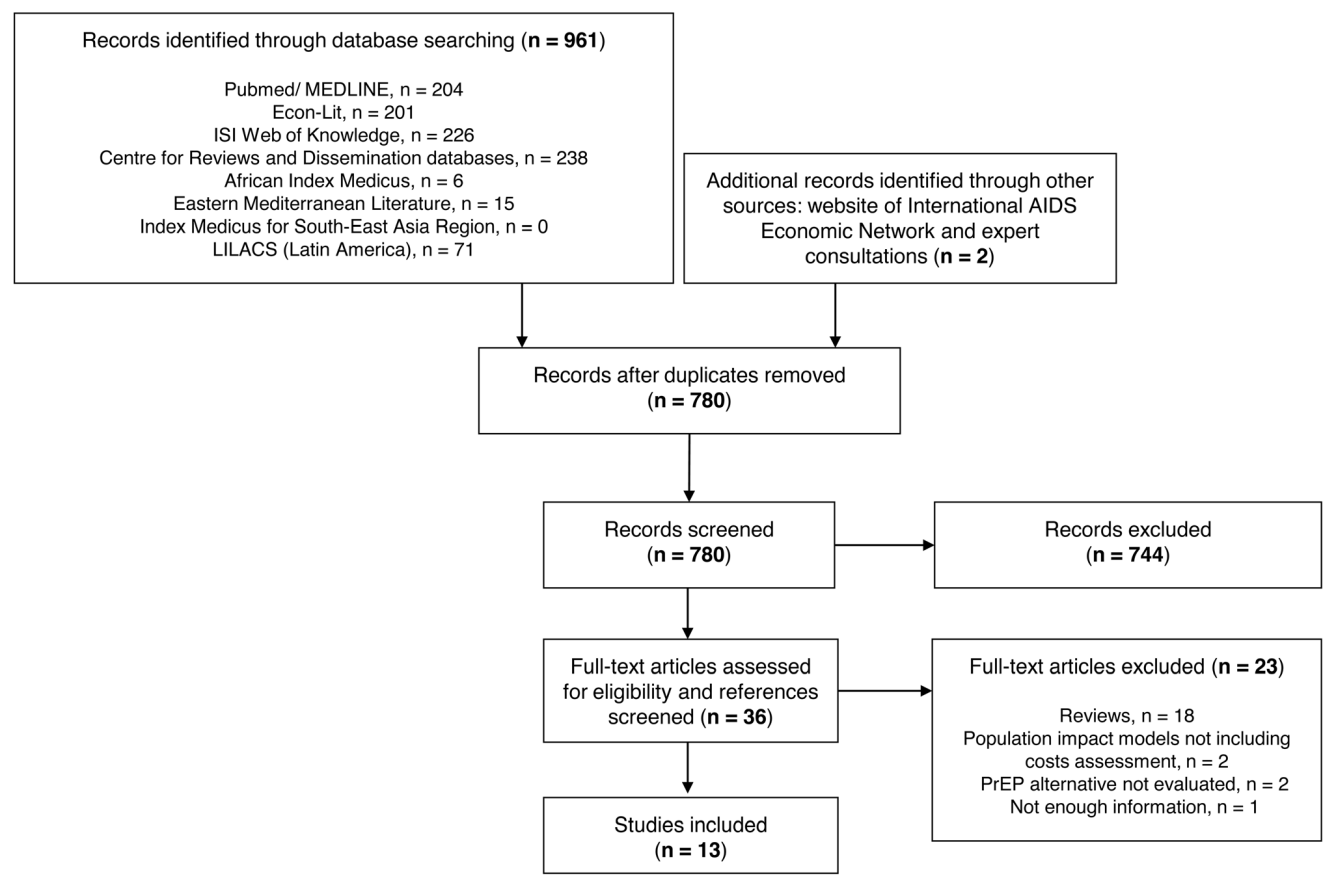
Flow diagram of study selection. Region-specific databases can be accessed as follows: African Index Medicus, http://indexmedicus.afro.who.int/; Eastern Mediterranean Literature, http://www.emro.who.int/; Index Medicus for South-East Asia Region, http://www.hellis.org/; LILACS, Latin America, http://www.bireme.br/iah2/homepagei.htm.

We screened 961 titles and abstracts retrieved from 14 electronic databases. After performing web searches and consulting experts in the field, 36 full text articles were evaluated. We also reviewed the reference lists and citations of these articles. Of these 36, 13 studies were included in the review [25]–[37]: 11 peer-reviewed publications and two peer-reviewed conference abstracts (Figure 1). Articles excluded are listed in Table S1 and a summary of conclusions of the articles included are presented in Table S2.

We present in Tables 1 to 4 the data extracted from the studies reviewed by study design, description of alternative programmes compared, impact, and cost assumptions. All studies were published between 2007 and 2013 and modelled the impact and cost, from a health care provider perspective, of PrEP scale-up in diverse settings. These settings included: heterosexual transmission in generalised epidemics in sub-Saharan Africa—the Southern Africa region [25], South Africa [28],[30],[31],[32],[36],[37]), and other modes of transmission in concentrated epidemics—among people who inject drugs (PWID) in Ukraine [33]; and men who have sex with men (MSM) in the USA [26],[29],[34],[35] and in Peru [27]. Timeframes varied from 5 to 20 y. All studies focused the models on high prevalence/incidence populations (Table 1).

**Table 1:** Study design.

Reference	Study Type	Setting/MoT	Population	Timeframe	HIV Incidence/Prevalence
**Generalised epidemics in southern Africa**					
Abbas [25]	Deterministic simulation; Risk heterogeneity by age, sex, sexual behaviour, and HIV drug resistance	Southern Africa/Heterosexual	15–49 y; General population	10 y	Prevalence: 20%^a^
Pretorius [30]	Deterministic simulation; Risk heterogeneity by age and sex	South Africa/Heterosexual	15–49 y; General population	10 y (programme scale-up: 5 y)	Prevalence: ±20% in 2008^b^
Hallett [28]	Microsimulation; Risk heterogeneity by age, sex, sexual behaviour, and conception intentions or pregnancy^c^	South Africa/Heterosexual	Serodiscordant couples	Each person is tracked until his/her 50th y	n/a
Williams [32]	Deterministic simulation; Risk heterogeneity not included	South Africa/Heterosexual	15–49 y; General population	From 2012 to 2020 (scale-up by 2015)	Prevalence: approximately 16% in 2012^b^
Walensky [31]	Monte Carlo state simulation; Risk heterogeneity by age	South Africa/Heterosexual	Women at higher risk	Each person is tracked until death	Incidence: <25 y, 2.2%; >25 y, 1.0%
Alistar [37]	Compartmental dynamic simulation Risk heterogeneity by sexual behaviour behaviour (number of partners and condom use)	South Africa/Heterosexual	15–49 y; General population	20 y	Initial prevalence in adults: 17.9% and initial incidence: 1.4%
Cremin [36]	Deterministic simulation;Risk heterogeneity by age, sex, male circumcision status, behavioural; risk (partner change rate, condom use)	South Africa/Heterosexual	15–54 y; General population	10 y (programme scale-up: 5 y)	Age- and sex-specific prevalence peaking at 30–44 y (women: >40% and 35–44 y men: >30%).
**Concentrated epidemics among MSM in high-income countries**					
Desai [26]	Stochastic simulation; Risk heterogeneity by age, sexual risk behaviour^d^	USA (NYC)/MSM	13–40 y; High risk MSM	5 y	Prevalence: 14.6% in 2008
Paltiel [34]	Monte Carlo state simulation; Risk heterogeneity by age (assumed higher incidence by age group)	USA/MSM	Average 34 y; High risk MSM	Each person is tracked until death	Incidence: 1.6% annual
Koppenhaver [29]	Compartmental dynamic simulation Risk heterogeneity not included	USA (urban)/MSM	13–40 y; All MSM	20 y	Prevalence: 17.5%
Juusola [35]	Deterministic simulation; Risk heterogeneity by sexual behaviour^e^	USA/MSM	13–64 y;	20 y	Prevalence: 12.3%; Incidence: 0.8% annual
**Concentrated epidemics among MSM in low- and middle-income countries**					
Gomez [27]	Deterministic simulation; Risk heterogeneity by sexual behaviour	Peru (Lima)/MSM	All MSM	10 y (programme scale-up: 5 y)	Incidence: MMSM, 1%; MMSW, 2.5%; SW, 3.1%; Trans, 7.3%
**Concentrated epidemics among PWID in low- and middle-income countries**					
Alistar [33]	Compartmental dynamic simulation Risk heterogeneity by IDU behaviour	Ukraine/IDU and heterosexual	15–49 y	20 y	Initial prevalence: 41.2% PWID, 1% general population

Study type refers to the type of model and the inclusion of risk heterogeneity in the population modelled. Setting/MoT refers to the geographical setting and the mode of transmission modelled.

a Female∶male ratio 1.66, based on data from urban antenatal care attendees in Zambia.

b Model initiated at a high prevalence then fitted to Department of Health data.

c Two types of couples were defined: (1) lower risk couples based on reported data from the Partners in Prevention HSV/HIV Transmission Study [49], and (2) couples at a higher risk reflecting a higher incidence. “Partners in Prevention” assumptions: incidence low (1.8/100 person-years at risk, high condom use); “more typical couples” assumptions: 50% of serodiscordant couples involved HIV-1 infected men. Compared to the partners in prevention cohort: condom use within the stable partnership was reduced by 25%, 50% more of the HIV-1 uninfected partners in couples had external partners, and frequency of unprotected sex with external partners was doubled.

d Very high risk was defined as a participant reporting unprotected sex in the last 6 mo or in exchange for money or drugs, anonymous sex, ≥5 sexual or needle sharing partners, and/or an STI diagnosis in the last 6 mo.

e The authors run the model separately for low risk and high risk populations. Therefore PrEP use in one group does not have an impact on the other (the mixing is considered totally assortative).

IDU, injection drug use; MMSM, men who mostly have sex with men; MMSW, men who mostly have sex with women; n/a, not applicable; SW, sex worker;

Trans, transgender or trans-sexual; USA (NYC), United States of America (New York City).

**Table 2:** Alternative programmes compared.

Reference	Base Comparison Scenario	PrEP Intervention		
		PrEP Regimen	Prioritisation	Coverage
**Generalised epidemics in southern Africa**				
Abbas [25]	No PrEP. ART was not modelled.	Once daily oral dosing	No prioritisation: general population. By sexual activity: two highest sexual activity groups prioritised. By age: 15–20 y group prioritised.	Percent of the population using PrEP: Optimistic scenario, 75%; Neutral scenario, 50%; Pessimistic scenario, 25%
Pretorius [30]	No PrEP. ART coverage expands at its current rate. ART efficacy: 90% reduction in transmission probability.	Once daily oral dosing	No prioritisation: 15–35 y; By age: 15–25 y, or 25–35 y	Percent of women using PrEP: 20%, dropout rate:1.5%
Hallett [28]	No PrEP. ART initiation for the infected partner when CD4 cell count fell below 200 cells/ml. In a separate scenario, expansion of eligibility criteria for ART initiation was included (below 350 CD4 cells/ml).	Once daily oral dosing	No prioritisation: Always use PrEP after diagnosis partner. By timing: Up to partner's ART init; up to partner's ART init+1 y; during conception/pregnancy^a^	Percent of the population using PrEP: see prioritisation
Williams [32]	No PrEP. The scale-up of ARV therapy was not modelled.	Vaginal gel, two doses pericoitally	PrEP used only by women	Percent of sex acts protected: High: 90%, Medium: 50%, Low: 25%
Walensky [31]	No PrEP. Patients identified as HIV infected received ART as per guidelines.	Vaginal gel, two doses pericoitally	PrEP used only by women. By age: ≤25 y (high inc. group)	Cohort-wide PrEP use continues until HIV infection or death.
Alistar [37]	No PrEP. 40% HIV infected patients received ART as per guidelines. ART efficacy: 95% reduction in transmission probability.	Once daily oral dosing	No prioritisation: general use; By sexual activity: groups of high number of partners and low condom use	Rate of recruitment into the program: 25%, 50%, 75%, 100%. Included a rate of dropout from PrEP.
Cremin [36]	ART efficacy: 96% reduction in transmission probability. Baseline scenarios varied: from status quo with current scale-up of ART to counterfactual including MC and ART scale-up. All scenarios included a 7/100 PY dropout rate while on ART.	Once daily oral dosing	No prioritisation: 15–54 y; By age: 15–24 y	Percent of the population group using PrEP: 40%, 80%
**Concentrated epidemics among MSM in high-income countries**				
Desai [26]	No PrEP. The scale-up of ARV therapy was not modelled.	Once daily oral dosing	No prioritisation—results not shown. Results for scenarios targeting high risk MSM only.	25% high risk^b^; (5.2% of all MSM)^c^; Discontinuation rate: 40% per year
Paltiel [34]	No PrEP. Patients identified as HIV infected received ART as per guidelines.	Once daily oral dosing	No prioritisation: all MSM. By age: <20 y.	Cohort-wide PrEP use continues until HIV infection or death.
Koppenhaver [29]	No PrEP. 25% of susceptible and undiagnosed MSM are tested per year, if eligible they start ART as per guidelines.	Once daily oral dosing	No prioritisation: all MSM.	100%
Juusola [35]	No PrEP. Patients identified as HIV infected received ART as per guidelines.	Once daily oral dosing	No prioritisation: all MSM; By sexual activity: high risk MSM.	100%, 50%, 20% of all MSM or those at high risk^d^
**Concentrated epidemics among MSM in low- and middle-income countries**				
Gomez [27]	No PrEP. Patients identified as HIV infected received ART as per guidelines (CD4<200 cells/ml) to achieve 40% coverage.	Once daily oral dosing	No prioritisation: uniform coverage. By sexual activity: some and high prioritisation.	Low 5%; High 20%
**Concentrated epidemics among PWID in low- and middle-income countries**				
Alistar [33]	No PrEP. Limited coverage of MMT and ART.	Once daily oral dosing	No prioritisation: all PWID: in all cases, MMT and PrEP are given only to PWID.	25%, 50% uninfected PWID. Included a rate of dropout from PrEP.

a Period trying to conceive and while pregnant.

b The authors also considered a scenario of 2.5% coverage, but explored results for the 25% scenario.

c Defined as those with more than five partners per y.

d Coverage includes only people fully adherent to PrEP.

init, initiation; MC, male circumcision; MMT, methadone maintenance treatment; n/a, not applicable.

**Table 3:** Impact: assumptions.

Reference	Effectiveness	Adherence	Behaviour Change (While on PrEP)	Resistance	Toxicity	DALY/QALY Assumptions
**Generalised epidemics in southern Africa**						
Abbas [25]	Effectiveness: product of efficacy and adherence level. Optimistic, 90%; Neutral, 60%; Pessimistic, 30%.	Doses are modelled to be missed at random.	Factor of increased rate of sex partner change for both infected and uninfected: from 1 to 2.	Secondary resistance selected by drug pressure in individuals infected by wild-type virus while on PrEP. Primary resistance acquired through transmission. Reversion after discontinuation of PrEP or transmission to a drug naive individual. Decreased transmission probability in drug-resistance individuals. Minor drug-resistance not transmitted. Re-emergence not included.	Not included.	n/a; Authors reported cost/infection averted only.
Pretorius [30]	Efficacy: reduction in acquisition probabilityOptimistic, 90%; Pessimistic, 70%	n/a	Optimistic, No change. Pessimistic, 30% decrease in condom use.	Not included.	Not included.	n/a; Authors reported cost/infection averted only.
Hallett [28]	Effectiveness: combination of “intrinsic efficacy” and adherence level. Based on PrEP trials of heterosexual couples. High, 80%; Low, 30%.	Varied with efficacy to obtain 30 and 80% effectiveness.	No change.	Not included.	Not included.	Primary outcome: cost/infection averted.QALY gained reported: Person-years lived weighted using utility (uninfected/infected, CD4 cell count, and treatment) [50]
Williams [32]	Efficacy: protection from HIV acquisition. Based on CAPRISA. High 60%; Medium 30%; Low 15%.	Modelled as percent of sexual acts protected.	n/a	Not included.	Not included.	One HIV infection averted saves 23 DALYs. Based on [51].
Walensky [31]	Effectiveness: reduction in HIV incidence. Based on CAPRISA: 39% [10–90]	Variation in effectiveness to include synergistic effects of increased condom use, adoption of MC, other behaviour changes, adherence and uptake of PrEP.	Variation in effectiveness to include synergistic effects of increased condom use, adoption of MC, other behaviour changes, adherence and uptake of PrEP.	PrEP users: 5% infected with ART-resistant virus. 10% decrease in the rate of virologic suppression of first-line ART for resistant patients.	Toxicity rate: 0.02% per mo, risk of death of 1/10,000 on PrEP.	n/a; Authors reported cost/infection averted only.
Alistar [37]	Effectiveness: 60% reduction in acquisition risk.	Adherence reflected in effectiveness.	No change.	Not included.	Not included.	QALY value for individuals in each compartment based on published literature. It varies by infection, disease stage and treatment status.
Cremin [36]	Efficacy as a reduction in acquisition of infection per PrEP protected sex act: 75%.	Proportion of a PrEP user's sex acts which benefit from PrEP: 95%.	No change.	Not included.	Not included.	QALY gained reported: Person-years lived weighted using utility (uninfected/infected, CD4 cell count, and treatment) [50]
**Concentrated epidemics among MSM in high-income countries**						
Desai [26]	Three mechanisms: (1) Basic: 50%–70% fully adherent; 0% otherwise; (2) Adherence: 50%–70% fully adherent; partial protection if partial adherence (30%–50%); (3) Exposure: 50%–70% moderate exposure; 30%–50% more exposure, fully adherent only.	Proportion of users with full adherence: High 95%, Medium 50%, or Low 30%	Population-wide increase of 0 to 20% in annual number of sexual partners.	Not included.	Not included.	QALY: Person-years lived weighted using utility (uninfected/infected, CD4 cell count, treatment) [50]
Paltiel [34]	Effectiveness: reduction in HIV incidence, 50% [10–90].	Variation in effectiveness to include synergistic effects of increased condom use, adoption of MC, other behaviour changes, adherence and uptake of PrEP.	Variation in effectiveness to include synergistic effects of increased condom use, adoption of MC, other behaviour changes, adherence and uptake of PrEP.	Scenarios: (1) All HIV-infected patients with a PrEP history are resistant. (2) No efavirenz-based regimen for patients with a history of PrEP. (3) Absolute 5% (0%–15%) decrease in rates of virologic suppression for all patients infected after PrEP.	(1) Chronic renal disease, 10% all PrEP patients, 10% reduction in quality of life; (2) 1% mortality at PrEP initiation.	Estimated quality of life for health states (based on CD4 cell count, HIV RNA level, relevant history).
Koppenhaver [29]	Effectiveness: reduction in incidence: 44%–73%.	Adherence reflected in effectiveness.	Not included.	Not included.	Not included.	Same as Desai [26].
Juusola [35]	Efficacy: Reduction in risk for infection, based on iPrEX: 44% [10–92].	100%^a^	Condom use (−20 to 20%), number of partners (−20 to 20%)	Not included.	Minor side effects (i.e., nausea included in SA).	Estimated quality of life for health states, adjusted utilities on average population age.
**Concentrated epidemics among MSM in low- and middle-income countries**						
Gomez [27]	Effectiveness: combination of “intrinsic efficacy” 92% [42–99] and adherence level. Based on iPrEX trial: 44% [15–63].	iPrEX adherence profile. Scenarios for more and less adherence included.	Variation in condom use while from −100 to +20%.	Not included.	Not included.	DALY averted/HIV infection averted calculated from average life expectancy and age at infection in Peru.
**Concentrated epidemics among PWID in low- and middle-income countries**						
Alistar [33]	Effectiveness: 60% reduction in acquisition risk (heterosexual) and 30% reduction in acquisition risk (needle-based)	Adherence reflected in effectiveness.	Not change.	Not included.	Not included.	QALY value for individuals in each compartment based on published literature. It varies by infection and disease stage, IDU status, and treatment status.

a Coverage includes only people fully adherent to PrEP.

IDU, injection drug users; init., initiation; MC, male circumcision; n/a, not applicable.

**Table 4:** Cost: assumptions (US$ in publication).

Reference	Drug Costs	Service Costs	Costs above Service Level	ART Costs	Discount Rate
**Generalised epidemics in southern Africa**					
Abbas [25]	Drug costs (2007 US$, per person-y): high, US$700; moderate, US$318; low, US$208.	Not included	Not included	Excluded costs of provision of ART.	Undiscounted
Pretorius [30]	Drugs (2010 US$): US$134 per person-y.	VCT, tests (serum creatinine, hepatitis B, pregnancy): US$16 per person-y.	Not included	Average cost of ART: US$600/person-y	Undiscounted^a^
Hallett [28]	Drugs and monitoring costs (2011 US$): US$150–US$250 per person-y. Includes lab testing, personnel, and drug costs.	See drug costs for description	Not included	Average cost of ART: US$450–US$800/person-y	3% annual discount rate
Williams [32]	Microbicide only (2010 US$): US$0.60 for two applications including gel, wrapping, and applicator.	Not included	Not included	Lifetime cost of ART: US$8,396 (for 23 y, approx. US$365/person-y)	Undiscounted^a^
Walensky [31]	Applicator and gel (2010 US$): US$0.32/dose, US$5/mo	HIV tests, chemistry panels (urea, creatinine, bilirubin, aspartate aminotransferase, alanine aminotransferase testing, and reagents, staff salary, equipment, overhead, and facilities): total US$188 per person-y.	See service costs for description	Average cost of ART: US$105–US$504/person-y	3% annual discount rate
Alistar [37]	Drugs (2012 US$): US$80	All individuals in the population incur an annual US$200 cost of general medical care.	Not included.	Average cost of ART: US$150/person-y. An additional US$1,000 per person-y in medical costs for HIV-infected individuals.	3% annual discount rate
Cremin [36]	Drugs (2012 US$): US$118 per person-y.	Testing (US$20), human resources (US$91), facilities (US$23).	Not included.	Average cost of ART: US$600/person-y	3% annual discount rate
**Concentrated epidemics among MSM in high-income countries**					
Desai [26]	Drugs (2007 US$): US$31/tablet; (US$11,315 per person-y)	Medical screening, ongoing medical monitoring and adherence promotion (1 mo after initiation and at 3-mo intervals): 1st year, US$1,300; each year after, US$1,020 per person/y.	Not included	Lifetime cost of ART: US$343,130	3% annual discount rate
	5-y combined cost for drug and support service: US$58,700 per person.	See drug costs for description			
Paltiel [34]	Drugs (2006US$): US$72–US$725/person-month; (US$864–US$8,700 per person-y)	Quarterly laboratory monitoring, semi-annual physical examinations, and annual full lipid panels: US$28 per person-month.	Not included	Excluded costs of provision of ART.	3% annual discount rate
	Approx. combined cost: US$1,200–US$9,036 per person-y.				
Koppenhaver [29]	Drugs (2010 US$): US$22 per person-day; (US$8,030 per person-y)	Quarterly HIV testing and monitoring for adverse events as gathered in Desai et al. [26].	Not included	Lifetime cost of ART: US$343,130	3% annual discount rate
Juusola [35]	Drugs (2010 US$): US$776 per person-month (approx. US$9,312 per person-y)	Based on CDC interim PrEP guidelines [9]: clinical screening, physician visits 5/y (HIV testing, risk-reduction counseling, condoms), testing for STI biannually, and renal function yearly: US$10,083 per person-y.	Not included	Average cost of ART: 15,589/person-y (for 23 y, US$365,470)	3% annual discount rate
**Concentrated epidemics among MSM in low- and middle-income countries**					
Gomez [27]	Drugs (2011 US$): US$420 and US$600 per person-y.	Based on CDC interim PrEP guidelines [9]. HIV screening, HIV testing every 3 mo during use, testing for renal function yearly, outreach and counselling services, condom and lubricant provision. US$525–US$830 per person-y.	Systemic costs at a 5% mark-up. Included in service costs.	Excluded costs of provision of ART. Cost of ART included in sensitivity analysis: US$1,000–US$3,000 per person-y.	3% annual discount rate
**Concentrated epidemics among PWID in low- and middle-income countries**					
Alistar [33]	High PrEP annual cost (US$2011): US$6,000	US$310 general medical costs, HIV medical costs are US$1,200.	Not included	ART costs US$450/y for general population, US$950 for PWID not in methadone, and US$750 for PWID in methadone, to account for counseling and additional efforts needed for IDUs	3% annual discount rate

approx, approximately; CDC, Centers for Disease Control and Prevention; n/a, not applicable/not available; person-y, person-year; VCT, voluntary counselling and testing.

a Assumed.

In all models, the comparison scenario did not include PrEP and assumptions varied regarding current treatment scale-up: from no ART programme included [25],[26],[32] to ART coverage remaining stable at a current level [27],[29],[31],[34],[35] or an ongoing ART programme coverage expansion [28],[30],[33],[36],[37]. While most of the studies looked at systemic PrEP (daily oral dosing), two studies in South Africa looked at vaginal gels [31],[32]. Coverage assumptions were stated as scenarios. The criteria used to characterise priority populations varied among the studies, including high risk of acquisition (defined by sexual activity, condom use, or HIV incidence) [25]–[27],[35],[37], age [25],[30],[31],[34],[36], and timing of PrEP use [28] in relation to users' life events (Table 2).

All models were transmission models, except for two Markov simulations [31],[34]. Efficacy and effectiveness estimates varied from estimated ranges that were assumed prior to the results from clinical trials and had wide ranges (from 10% to 90%) [25],[26],[30],[33],[34] to estimates available directly from clinical trials [27]–[29],[31],[32],[35]–[37]. Several authors interpreted effectiveness as being dependent on the product efficacy and the individual-level adherence, specifically modelling this interaction [25]–[29],[32],[36]. Adherence assumptions varied from random [25] to profiles based on observations from published trials [27]–[29],[32]. Potential behaviour change following the introduction of a PrEP programme was included in the models as an increase in the number of sexual partners [25],[26], changes in condom use [27],[30], or both [35]. Drug resistance associated with PrEP use was explicitly modelled in one study [25], while in two further studies it was represented as a decrease in the rate of virologic suppression while on subsequent treatment [31],[34]. The studies did not assume any reduction in the quality of life or disability weights due to PrEP use, with the exception of three studies where toxicity to PrEP was addressed through a reduction in the quality of life and/or an excess fatality rate among PrEP users (Table 3) [31],[34],[35].

The majority of studies presented costs for PrEP programmes including drugs and monitoring costs, except for two studies that included drug costs only [25],[32]. Costs above service level were only included in two studies, as overheads [31] or a mark-up percentage of 5% [27]. Overall PrEP programme costs were consistent among studies by setting and ranged from high in the USA (between US$8,000 and US$12,000 per person-year) to low in South Africa (between US$80 to US$250). All cost estimates were driven by the cost of drugs. Three studies in the USA [26],[29],[35] and six in South Africa [28],[30]–[32],[36],[37] included averted ART costs. Ranges of estimated cost of ART were consistent among studies and context-specific (<US$1,000 per person-year in South Africa to >US$15,000 per person-year in the USA) (Table 4).

We present all cost-effectiveness estimates in Table 5 by epidemiological context and scenario modelled.

**Table 5:** Cost-effectiveness estimates by scenario.

Reference	Scenario Description: Prioritisation	Estimate		
		Measure	US$ in Publication	2012US$
**Generalised epidemics in southern Africa**				
Abbas [25]	Pessimistic: high sexual activity group	Cost/infection averted	2,949–9,923	3,450–11,609
	Pessimistic: 15–20 y	Cost/infection averted	20,202–67,970	23,636–79,525
	Pessimistic: no prioritisation	Cost/infection averted	20,164–67,842	23,591–79,375
	Neutral: high sexual activity group	Cost/infection averted	1,160–3,904	1,357–4,567
	Neutral: 15–20 y	Cost/infection averted	8,968–30,173	10,492–35,302
	Neutral: no prioritisation	Cost/infection averted	9,629–32,398	11,265–37,905
	Optimistic: high sexual activity group	Cost/infection averted	638–2,147	746–2,512
	Optimistic: 15–20 y	Cost/infection averted	5,723–19,254	6,695–22,527
	Optimistic: no prioritisation	Cost/infection averted	6,812–22,918	7,970–26,814
Pretorius [30]	Optimistic: women 15–25 y, no behaviour change	Cost/infection averted	>25,000	>26,625
	Optimistic: women 15–35 y, no behaviour change	Cost/infection averted	>22,500	>23,963
	Optimistic: women 25–35 y, no behaviour change	Cost/infection averted	>20,000	>21,300
	Medium efficacy: women 25–35 y, behaviour change	Cost/infection averted	>30,000	>31,950
Hallett [28]	Efficacy range, high risk: conception or pregnancy use	Cost/infection averted	−6,000 to 8,000	−6,192 to 8,256
	Efficacy range, low risk: conception or pregnancy use	Cost/infection averted	−2,000 to 12,000	−2,064 to 12,384
	Efficacy range, high risk: up to ART initiation	Cost/infection averted	−2,200 to 21,000	−2,270.4 to 21,672
	Efficacy range, high risk: always use PrEP	Cost/infection averted	0–26,000	0–26,832
	Efficacy range, low risk: always use PrEP	Cost/infection averted	6,000–66,000	6,192–68,112
	Optimistic, low risk, high ART cost: up to ART initiation	Cost/infection averted	3,000	3,096
	Optimistic, low risk, high ART cost: up to ART initiation +1 y	Cost/infection averted	3,000	3,096
	Optimistic, high risk: up to ART initiation	Cost/QALY gained	−200 to 500	−**206** a **to 516** a
	Optimistic, low risk: up to ART initiation	Cost/QALY gained	260–1,600	**268** a **–1,651** a
	Pessimistic, high risk: up to ART initiation	Cost/QALY gained	700–1,900	**722** a **–1,960** a
	Pessimistic, low risk: up to ART initiation	Cost/QALY gained	2,500–4,900	**2,580** a **–5,056** a
Williams [32]	CAPRISA efficacy: high coverage	Cost/infection averted	420–2,982	447–3,175
	CAPRISA efficacy: low coverage	Cost/infection averted	562–4,222	598–4,496
	CAPRISA efficacy: high coverage	Cost/DALY averted	18–130	**19** a **–138** a
	CAPRISA efficacy: low coverage	Cost/DALY averted	27–181	**28** a **–193** a
Walensky [31]	CAPRISA efficacy, test freq 3 mo: high incidence women	Cost/life year saved	1,600	**1,704** a
	CAPRISA efficacy, test freq 1 mo: high incidence women	Cost/life year saved	2,700	**2,876** a
Alistar [37] b	PrEP: no prioritisation recruitment rate 25% to 100%, no ART expansion	Cost/QALY gained	1,200	**1,200** a
	PrEP: high risk group recruitment rate 50% to 100%, no ART expansion	Cost/QALY gained	CS	**CS** a
	PrEP: no prioritisation recruitment rate 25% to 100%, ART +25% as per guidelines	Cost/QALY gained	980–1,050	**980** a **–1,050** a
	PrEP: high risk group recruitment rate 100%, ART +25% as per guidelines	Cost/QALY gained	50	**50** a
	PrEP: no prioritisation recruitment rate 25% to 100%, ART +50% as per guidelines	Cost/QALY gained	900–1,000	**900** a **–1,000** a
	PrEP: high risk group recruitment rate 100%, ART +50% as per guidelines	Cost/QALY gained	160	**160** a
	PrEP: no prioritisation recruitment rate 25% to 100%, ART +75% as per guidelines	Cost/QALY gained	860–970	**860** a **–970** a
	PrEP: high risk group recruitment rate 100%, ART +75% as per guidelines	Cost/QALY gained	210	**210** a
	PrEP: no prioritisation recruitment rate 25% to 100%, ART +100% as per guidelines	Cost/QALY gained	840–950	**840** a **–950** a
	PrEP: high risk group recruitment rate 100%, ART +100% as per guidelines	Cost/QALY gained	230	**230** a
	PrEP: no prioritisation recruitment rate 25% to 100%, universal ART +25%	Cost/QALY gained	810–940	**810** a **–940** a
	PrEP: high risk group recruitment rate 100%, universal ART +25%	Cost/QALY gained	220	**220** a
	PrEP: no prioritisation recruitment rate 25% to 100%, universal ART +50%	Cost/QALY gained	760–900	**760** a **–900** a
	PrEP: high risk group recruitment rate 100%, universal ART +50%	Cost/QALY gained	280	**280** a
	PrEP: no prioritisation recruitment rate 25% to 100%, universal ART +75%	Cost/QALY gained	740–890	**740** a **–890** a
	PrEP: high risk group recruitment rate 100%, universal ART +75%	Cost/QALY gained	290	**290** a
	PrEP: no prioritisation recruitment rate 25% to 100%, universal ART +100%	Cost/QALY gained	740–880	**740** a **–880** a
	PrEP: high risk group recruitment rate 100%, universal ART +100%	Cost/QALY gained	300	**300** a
Cremin [36] c	PrEP: no prioritisation, cov 4.4% of 15–54 y (baseline: status quo, current ART scale-up)	Cost/infection averted	9,390	9,390
	PrEP: prioritisation, cov 7.3% of 15–24 y (baseline: status quo, current ART scale-up)	Cost/infection averted	10,540	10,540
	No PrEP, 80% universal ART (baseline: 80% ART200 and 80% MC)	Cost/infection averted	10,530	10,530
	PrEP: 15–24 y cov 40%, 80% universal ART (baseline: 80% ART200, 80% MC, 80% ART350)	Cost/infection averted	39,900	39,900
	PrEP: 15–54 y cov 80%, 80% universal ART (baseline: 80% ART200, 80% MC)	Cost/infection averted	20,500	20,500
**Concentrated epidemics among MSM in high-income countries**				
Desai [26] d	Exposure, pessimistic: high adherence	Cost/QALY gained	6,661–36,268	**7,793** e **–42,433** e
	Exposure, pessimistic: medium adherence	Cost/QALY gained	55,167–84,774	**64,545** f **–99,185** f
	Exposure, pessimistic: low adherence	Cost/QALY gained	113,601–143,208	**132,913** f–167,553
	Adherence, pessimistic: high adherence	Cost/QALY gained	CS–8,158	**CS** e **–9,545** e
	Adherence, pessimistic: medium adherence	Cost/QALY gained	CS–10,327	**CS** e **–12,082** e
	Adherence, pessimistic: low adherence	Cost/QALY gained	CS–13,499	**CS** e **–15,793** e
	Basic, pessimistic: high adherence	Cost/QALY gained	CS–15,099	**CS** e **–17,665** e
	Basic, pessimistic: medium adherence	Cost/QALY gained	17,168–46,775	**20,086** e **–54,726** f
	Basic, pessimistic: low adherence	Cost/QALY gained	66,896–96,502	**78,268** f **–112,907**
	Exposure, optimistic: high adherence	Cost/QALY gained	CS–9,925	**CS** e **–11,612** e
	Exposure, optimistic: medium adherence	Cost/QALY gained	13,307–42,914	**15,569** e **–50,209** f
	Exposure, optimistic: low adherence	Cost/QALY gained	46,502–76,109	**54,407** f **–89,047** f
	Adherence, optimistic: high adherence	Cost/QALY gained	CS	**CS** e
	Adherence, optimistic: medium adherence	Cost/QALY gained	CS	**CS** e
	Adherence, optimistic: low adherence	Cost/QALY gained	CS	**CS** e
	Basic, optimistic: high adherence	Cost/QALY gained	CS–1,009	**CS** e **–1,180** e
	Basic, optimistic: low adherence	Cost/QALY gained	37,947–67,553	**44,398** e **–79,037** f
	Basic, optimistic: medium adherence	Cost/QALY gained	CS–28,393	**CS** e **–33,220** e
Paltiel [34]	Medium efficacy: no prioritisation	Cost/QALY gained	298,000	359,984
	High efficacy: no prioritisation	Cost/QALY gained	107,000	**129,256** f
	Medium efficacy, low cost	Cost/QALY gained	114,000	**137,712** f
	Medium efficacy: young	Cost/QALY gained	189,000	228,312
Koppenhaver [29]	High adherence: no prioritisation	Cost/QALY gained	353,739	376,732
	iPrEX adherence: no prioritisation	Cost/QALY gained	570,273	607,341
Juusola [35]	Cov 100%, PrEP cost US$26/d, no resistance: high risk MSM	Cost/QALY gained	52,443	**55,852** f
	Cov100%, PrEP cost US$26/d, no resistance: no prioritisation	Cost/QALY gained	216,480	230,551
	Cov 100%, high eff, PrEP cost US$26/d, no resistance: high risk MSM	Cost/QALY gained	35,080	**37,360** e
	Cov 100%, high eff, PrEP cost US$26/d, no resistance: no prioritisation	Cost/QALY gained	146,228	155,733
	Cov 100%, PrEP cost US$15/d, no resistance: no prioritisation	Cost/QALY gained	131,277	**139,810** f
	Cov 100%, PrEP cost US$50/d, no resistance: high risk MSM	Cost/QALY gained	104,516	**111,310** f
	Cov 100%, PrEP cost (50% ARV), no resistance: high risk MSM	Cost/QALY gained	25,165	**26,801** e
	Cov 100%, PrEP cost (75% ARV), no resistance: high risk MSM	Cost/QALY gained	38,804	**41,326** e
	Cov 100%, no resistance, 8% reduction QoL: high risk MSM.	Cost/QALY gained	95,006	**101,181** f
	Cov 100%, PrEP cost US$26/d, resistance: high risk MSM	Cost/QALY gained	57,861	**61,622** f
	Cov 100%, PrEP cost US$26/d, resistance: no prioritisation	Cost/QALY gained	233,040	248,188
	Cov 50%, PrEP cost US$26/d, no resistance: high risk MSM	Cost/QALY gained	44,556	**47,452** e
	Cov50%, PrEP cost US$26/d, no resistance: no prioritisation	Cost/QALY gained	188,421	200,668
	Cov 50%, high eff, PrEP cost US$26/d, no resistance: high risk MSM	Cost/QALY gained	26,766	**28,506** e
	Cov 50%, high eff, PrEP cost US$26/d, no resistance: no prioritisation	Cost/QALY gained	120,080	**127,885** f
	Cov 50%, PrEP cost US$15/d, no resistance: no prioritisation	Cost/QALY gained	113,935	**121,341** f
	Cov 50%, PrEP cost US$50/d, no resistance: high risk MSM	Cost/QALY gained	89,658	**95,486** f
	Cov 50%, PrEP cost (50% ARV), no resistance: high risk MSM	Cost/QALY gained	20,930	**22,290** e
	Cov 50%, PrEP cost (75% ARV), no resistance: high risk MSM	Cost/QALY gained	32,743	**34,871** e
	Cov 50%, no resistance, 8% reduction QoL: high risk MSM.	Cost/QALY gained	72,762	**77,492** f
	Cov 50%, PrEP cost US$26/d, resistance: high risk MSM	Cost/QALY gained	56,492	**60,164** f
	Cov 50%, PrEP cost US$26/d, resistance: no prioritisation	Cost/QALY gained	226,325	241,036
	Cov 20%, PrEP cost US$26/d, no resistance: high risk MSM	Cost/QALY gained	40,279	**42,897** e
	Cov20%, PrEP cost US$26/d, no resistance: no prioritisation	Cost/QALY gained	172,091	183,277
	Cov 20%, high eff, PrEP cost US$26/d, no resistance: high risk MSM	Cost/QALY gained	22,374	**23,828** e
	Cov 20%, high eff, PrEP cost US$26/d, no resistance: no prioritisation	Cost/QALY gained	105,066	**111,895** f
	Cov 20%, PrEP cost US$15/day, no resistance: no prioritisation	Cost/QALY gained	103,841	**110,591** f
	Cov 20%, PrEP cost US$50/d, no resistance: high risk MSM	Cost/QALY gained	81,593	**86,897** f
	Cov 20%, PrEP cost (50% ARV), no resistance: high risk MSM	Cost/QALY gained	18,637	**19,848** e
	Cov 20%, PrEP cost (75% ARV), no resistance: high risk MSM	Cost/QALY gained	29,458	**31,373** e
	Cov 20%, no resistance, 8% reduction QoL: high risk MSM	Cost/QALY gained	62,431	**66,489** f
	Cov 20%, PrEP cost US$26/d, resistance: high risk MSM	Cost/QALY gained	78,884	**84,011** f
	Cov 20%, PrEP cost US$26/d, resistance: no prioritisation	Cost/QALY gained	303,091	322,792
**Concentrated epidemics among MSM in low- and middle-income countries**				
Gomez [27]	Low coverage: high prioritisation	Cost/DALY averted	403–637	**415** g **–657** g
	Low coverage: some prioritisation	Cost/DALY averted	447–707	**461** g **–729** g
	Low coverage: no prioritisation	Cost/DALY averted	1,076–1,702	**1,110** g **–1,756** g
	High coverage: high prioritisation	Cost/DALY averted	665–1,052	**686** g **–1,085** g
	High coverage: some prioritisation	Cost/DALY averted	886–1,400	**914** g **–1,445** g
	High coverage: no prioritisation	Cost/DALY averted	1,125–1,779	**1,161** g **–1,835** g
**Concentrated epidemics among PWID in low- and middle-income countries**				
Alistar [33]	MMT 25%, no PrEP	Cost/QALY gained	530	**546** h
	MMT 25%, ART 80% (for IDU and general population), no PrEP	Cost/QALY gained	870	**896** h
	MMT 25%, ART 80% (for IDU and general population), PrEP 25% to 50%	Cost/QALY gained	3,080–3,910	**3,172** h **–4,027** i
	PrEP 25% to 50%	Cost/QALY gained	14,590–14,680	15,028–15,120
	MMT 25%, PrEP 25% to 50%	Cost/QALY gained	4,800–6,100	**4,944** i **–6,283** i
	ART 80% (for IDU and general population), PrEP 25% to 50%	Cost/QALY gained	3,290–4,210	**3,389** h **–4,336** i

Thresholds used to determine cost-effectiveness, based on World Bank database [23]. Bold-black signifies an estimate is cost-effective or very cost-effective with regards to the country-specific threshold.

a For South Africa, an intervention is considered very cost-effective at a threshold of less than 1× GDP per capita, US$8,070.

b In Alistar et al., several scenarios were considered for ART recruitment rates of 25%, 50%, 75%, and 100% in addition to the 40% status quo coverage as per guidelines and following universal access.

c In Cremin et al., several scenarios were considered for ART coverage. ART200: coverage of ART in HIV-infected people starting at CD4 count of <200 cells/ml; ART350: coverage of ART in HIV-infected people starting at CD4 count of <350 cells/ml; universal ART: coverage of ART in HIV-infected people starting at any CD4 count level.

d In Desai et al., the authors considered three effectiveness mechanisms: basic, adherence-dependent, and exposure-dependent.

e For USA, an intervention is considered very cost-effective at a threshold of less than 1× GDP per capita, US$48,442.

f For USA, an intervention is considered cost-effective between 1× GDP per capita, US$48,442 and 3× GDP per capita, US$145,326.

g For Peru, an intervention is considered very cost-effective at a threshold of less than 1× GDP per capita, US$ US$6,009.

h For Ukraine, an intervention is considered very cost-effective at a threshold of less than 1× GDP per capita, US$3,615.

i For Ukraine, an intervention is considered cost-effective between 1× GDP per capita, US$3,615 and 3× GDP per capita, US$10,845.

cov., coverage; CS, cost saving; freq, frequency; MC, male circumcision; MMT, methadone maintenance treatment; QoL, quality of life; resist., resistance.

### Generalised Epidemics in Southern Africa (*n* = 7)

Studies on topical PrEP and two studies on oral PrEP suggest the intervention to be cost-effective (topical PrEP: <200 US$/DALY [32], <3,000 US$/LYS [31]; oral PrEP: <5,000 US$/QALY [28], <2,800 US$/QALY [37]) using benchmarks for cost-effectiveness specific to South Africa [22]. Three studies reported cost/infection averted only, estimates ranging from US$1,000 to 39,900 [25],[30],[36].

For topical PrEP, the two studies presented different estimates of cost-effectiveness: less cost-effective in Walensky et al. [31] (<US$1,600–US$2,700/life year saved) than in Williams et al. [32] (<US$18–US$181/DALY averted) due to a more comprehensive set of assumptions in the former (i.e., inclusion of the above service level costs of providing PrEP, adverse outcomes, topical PrEP toxicity, and resistance as well as a lifelong use of PrEP and discounting) [31],[32]. Prioritisation to high-risk key populations (high incidence groups, such as young women in South Africa) and improvements in adherence maximised the effectiveness of a topical PrEP programme [31],[32].

For oral PrEP, the impact was estimated to be higher if PrEP was prioritised for use among people at higher risk of HIV acquisition compared to no prioritisation strategy in four of the five studies included (i.e., higher sexual activity groups in Abbas et al. [25], couples at higher risk in Hallett et al. [28], people with high number of partners and low condom use in Alistar et al. [37], and younger women in Pretorius et al. [30]). In Cremin et al. [36], the authors compared PrEP prioritised to 15 to 24 years old to no prioritisation (PrEP available to the total adult population: 15 to 54 years old) and found the impact of the two strategies was very similar. However, the group prioritised (15 to 24 years old) did not present the highest risk of infection in this population. The impact of prioritising by age may be more evident when the intervention is aimed at age groups where incidence peaks (in this case among the 25- to 34-year age group).

Four studies analysed the interactions between oral PrEP and an expanding ART programme. Pretorius et al. found oral PrEP cost-effectiveness and its impact at population level to be considerably reduced if PrEP is added to the expanding ART programme [30]. Accordingly, Alistar et al. and Cremin et al. found that expanding ART coverage in this setting will be the more attractive strategy than investing in oral PrEP [36],[37]. However, Alistar et al. found PrEP to be cost saving, when delivered to individuals at greater risk of infection with no ART expansion [37]. Cremin et al. [36] found that a PrEP intervention was not cost-saving when implemented on top of a base case that included an 80% coverage of ART for people with CD4 counts of less than 200 cells/ml and male circumcision to be scaled up to 80%. Hallett et al. compared early ART to PrEP in HIV-serodiscordant couples, finding that if higher risk couples change their behaviour (for example through risk reduction counselling), earlier initiation of ART might become a cost-effective alternative to oral PrEP [28]. Assumptions about behavioural change (an increase in the number of partners) was a key driver of cost-effectiveness in Abbas et al. [25], while Pretorius et al. [30] found a lesser impact on cost-effectiveness following changes in condom use. This might be explained by the inclusion in Pretorius et al. of a background decrease in condom use with age, with older women tending to have less condom use. Hallett et al. did not include changes in behaviour due to oral PrEP introduction in their analyses. Resistance and toxicity levels did not significantly affect cost-effectiveness estimates [25],[31].

### Concentrated Epidemics among MSM in the USA (*n* = 4)

Pre-iPrEX modelling studies estimated cost-effectiveness of PrEP interventions among MSM with mixed results in the USA. The cost per QALY gained presented by Paltiel et al. [34] was considerably higher (US$298,000/QALY gained) than that presented by Desai et al. [26] (from cost saving to a maximum of US$143,208/QALY gained). This difference is primarily due to the inclusion of benefits from reduced onward transmission in the latter. The authors of post-iPrEX studies are in agreement that PrEP use among populations of MSM in the USA could have a significant impact on the domestic HIV epidemic. However, Koppenhaver et al., while exploring only scenarios with no prioritisation, found a PrEP intervention not to be cost-effective [29]. Juusola et al. found PrEP to be cost-effective under certain assumptions (i.e., prioritisation scenarios and no prioritisation scenarios including high product effectiveness or low drug costs [US$15/day for oral PrEP to the equivalent of 50% or 75% the cost of ART]) [35]. Both studies concluded that a PrEP programme might not be affordable due to the high cost of drugs used for PrEP (US$8,000 to US$9,300 per person-years for PrEP drugs only) [29],[35]. In this setting, the benefits of PrEP were expected to be offset by relatively small increases in the number of partners in one study [26]. Conversely, resistance was not found to have a strong impact on cost-effectiveness estimates [34],[35]. Varying levels of toxicity to PrEP had the potential to counterbalance PrEP benefits in two studies [34],[35].

### Concentrated Epidemics among MSM in Peru (*n* = 1)

PrEP could be a cost-effective addition to current prevention programmes in Peru for MSM populations (up to US$1,702/DALY averted) using benchmarks for cost-effectiveness specific to Peru [22]. However, even if PrEP drugs are expected to cost less than in settings such as the USA, a PrEP programme in this middle-income country might well require significant expenditure [27]. Behaviour change was not estimated to significantly affect cost-effectiveness estimates. It would result in detrimental effects (increases in the number of infections) only if PrEP efficacy and adherence were both assumed to be low [27]. The effect of prioritisation appears to be less pronounced in those scenarios with high coverage levels where saturation of coverage of those at highest risk occurs early during implementation and higher levels of coverage of lower-risk populations is achieved [27].

### Concentrated Epidemics among People Who Inject Drugs in Ukraine (*n* = 1)

Alistar et al. [33] found PrEP not to be a cost-effective intervention in isolation from other HIV control interventions for use among populations of PWID (US$14,590–US$14,680/QALY gained) using benchmarks for cost-effectiveness specific to Ukraine [22]. PrEP is considerably less attractive when compared to the expansion of either methadone maintenance therapy (US$530/QALY gained) or to the combination of methadone maintenance therapy and ART for those in need (US$870/QALY gained) [33].

## Discussion

This systematic review included 13 modelling studies estimating the cost and potential population-level impact of introducing a PrEP programme in generalised and concentrated epidemic settings. Our findings show that PrEP is estimated to have the potential to be a cost-effective addition to HIV prevention programmes in some settings. However, the cost-effectiveness of PrEP is likely to depend on considerations such as cost, the epidemic context, PrEP programme coverage and prioritisation strategies, as well as individual adherence levels and PrEP efficacy estimates.

To prevent heterosexual transmission in the generalised epidemics of southern Africa, PrEP is potentially a cost-effective intervention. Topical PrEP, in particular, could have a significant impact in South Africa, providing a much-needed female-initiated prevention option. However, it should be noted that funding PrEP while other potentially more cost-effective HIV prevention interventions remain under-funded may have high opportunity costs. In concentrated epidemics, such as MSM-driven epidemics both in Peru and in the USA, PrEP could have a substantial impact on the epidemic but may not be affordable at current drug prices. In Ukraine, expansion of ART coverage and methadone maintenance treatment programmes for PWID should be a first priority, with PrEP potentially added on within a combination prevention framework. However, evidence to date shows PrEP might not be cost-effective in this setting at current drug prices. Nevertheless, findings from the phase III Bangkok Tenofovir Study of PrEP among PWID will shed light on the efficacy estimates of PrEP in this population and inform future model estimates in similar epidemic contexts [38].

In all settings, the price of drugs is a limiting factor in terms of affordability of PrEP programmes as has been previously suggested [39],[40], and is key to determining cost-effectiveness. Moreover, the findings above predominately exclude important service and above service costs of providing PrEP (i.e., regular HIV testing and blood chemistry panels; the costs of possible adverse outcomes, including PrEP-related toxicity and potential drug resistance attributable to PrEP; and system-wide costs of implementing a PrEP programme). All of these should be considered to improve the validity and utility of estimates. Another key limitation among the studies is that the majority did not include savings in treatment and hospitalisation due to secondary infections averted. Although carrying significant uncertainties, the inclusion of these benefits allows a more informed consideration of potential PrEP benefits within broader programmatic planning for HIV prevention and care.

In the models reviewed, several prioritisation strategies were explored. Prioritisation by sexual activity characteristics to deliver PrEP to those populations at highest risk of HIV exposure improved the cost-effectiveness estimates. However, the extent to which prioritising populations at higher risk improves cost-effectiveness results in the models depends largely on the assumptions made about sexual mixing and risk heterogeneity. Extra costs related to the identification and engagement of priority populations were not included in any of the studies, neither were considerations in terms of economies of scale. Furthermore, as results from the enrolment phase of iPrEX Ole show (65% of trial participants decided to continue taking PrEP), not all individuals at higher risk are willing to use PrEP [41]. Identifying and meaningfully engaging those at highest risk in tailored HIV prevention strategies represents a significant challenge for decision makers, health care providers, and prevention advocates.

Prioritisation by age was a strategy advanced in several studies. In the studies reviewed, prioritisation by age group resulted in a lower cost-effectiveness benefit compared to prioritisation strategies based on self-reported risk behaviour [25],[30],[31],[34]. However, the former has the advantage of being straightforward to implement compared to a selection of potential PrEP users based on self-reported risk behaviour. In contexts such as South Africa and the USA, age prioritisation clearly would focus on those populations at higher risk of HIV acquisition. Another prioritisation strategy analysed in one study was the delivery of PrEP depending on the stage in users' lives. In reality, PrEP use will not need to be sustained throughout an individual's lifetime but may vary as his or her risk situation changes over time. People may opt to use PrEP during specific higher risk life periods, such as during periods of active sex work or when serodiscordant couples are trying to conceive a child [42],[43]. Understanding potential scenarios of PrEP use over the life cycle is essential for decision makers to be able to evaluate the possible impact of PrEP programmes in their local contexts. An additional consideration concerns intermittent PrEP. The first report of safety and adherence to an intermittent PrEP regimen in Kenya showed that among MSM and female sex workers adherence was lower than for daily dosing [44]. Results from two phase II trials underway in France [45] and the USA [46] will help inform adherence requirements and, should intermittent pre- and post-exposure dosing be proven effective, help tailor PrEP programmes to consumer demand [47].

Behavioural change due to PrEP use and adherence to PrEP were estimated to have potentially significant impacts on programme effectiveness. While the emergence of drug resistance due to PrEP programme scale-up and PrEP-related toxicity assumptions did not significantly affect cost-effectiveness estimates, improvement of drug resistance surveillance systems as well as effective adherence counselling will be essential components of PrEP programme implementation, in addition to behavioural counselling.

This review has several limitations. The geographical coverage of the studies reviewed is partial and both the impact and cost evaluations are highly setting-specific, limiting the generalisability of the findings. We were unable to perform a meta-analysis due to the variability across studies in reporting outcomes. Nevertheless, in order to compare cost-effectiveness estimates across settings, we used the thresholds proposed by the WHO-CHOICE Project and the Commission on Macroeconomics as a benchmark [22]. These standards are based on the GDP per capita, assuming that a society is willing to pay the equivalent of up to one GDP per capita (for highly cost-effective interventions) or between one and three times the GDP per capita (for a cost-effective intervention for a DALY averted, QALY saved, or LYS). This is a normative selection of cost-effectiveness thresholds, albeit regarded as useful from a decision analytic perspective [24].

It is worth noting that, with the exception of four studies in South Africa [28],[30],[36],[37], research comparing the potential trade-offs of earlier treatment for prevention versus PrEP remains an important gap in the literature that should be addressed, especially in concentrated epidemics. Cost-effectiveness studies that demonstrate where resources applied can have the greatest impact will help inform this complicated decision-making, but these are not the only considerations. The decision to include a PrEP option within the combination prevention package requires input from all strata of society. For instance, in contexts where universal access to ART for patients in need has not been achieved, PrEP programme planning processes will be challenged by concerns about social justice, equity, and affordability. This is in addition to the hurdles of overcoming the marginalisation, stigmatisation, and criminalisation of many of the populations that would most benefit from tailored HIV prevention programming that includes the choice of PrEP. Disentangling these issues will be critical for effective decision-making, as will the consideration of potential synergies between an expanded testing and treatment programme and a PrEP programme.

While the interest of donors for modelling studies that compare the cost-effectiveness of different HIV prevention methods is expected to increase [48], current evidence is already available to aid policy makers in assessing PrEP as a new prevention option. In this context, our review sheds light on the main considerations that decision makers need to address when judging the relevance of cost-effectiveness estimates of a potential PrEP programme and the potential gaps in the modelling evidence. Given that our review shows that setting and target population are critical drivers of cost-effectiveness, the next step is to conduct context-specific demonstration studies, including comprehensive cost analyses, of different prioritisation and adherence promotion strategies to ensure that the maximum benefit from the introduction of PrEP is realised within combination HIV prevention programmes.

## Supporting Information

Table S1 **List of publications reviewed for inclusion and excluded from the review.**
(DOCX)

Table S2 **Summary of conclusions of articles included in the review.**
(DOCX)

Text S1 **PRISMA checklist.**
(DOCX)

Text S2 **Protocol for systematic review (version 2).**
(PDF)

## Abbreviations

ART: antiretroviral treatment
ARV: antiretroviral
DALY: disability-adjusted life year
GDP: gross domestic product
LYS: life-year saved
MSM: men who have sex with men
PrEP: pre-exposure prophylaxis
PWID: people who inject drugs
QALY: quality-adjusted life year
TDF/FTC: tenofovir/emtricitabine
