# Supplementary material for: The Cost and Impact of Scaling Up Pre-exposure Prophylaxis for HIV Prevention: A Systematic Review of Cost-Effectiveness Modelling Studies
Source: PLoS Med. 2013 Mar 12;10(3):e1001401. doi: 10.1371/journal.pmed.1001401 (PMC3595225; doi:10.1371/journal.pmed.1001401)
Supplement: Table S1 — List of publications reviewed for inclusion and excluded from the review. (DOCX) [file pmed.1001401.s001.docx]

**The cost and impact of scaling-up pre-exposure prophylaxis for HIV prevention: a systematic review of cost-effectiveness modelling studies.** Gomez GB, Borquez A, Case KK, Wheelock A, Vassall A, Hankins (**SUPPLEMENTARY MATERIAL - results)**

**Table S1:** List of publications reviewed for inclusion and excluded from the review.

| Reference | Reason for exclusion |
| --- | --- |
| Abbas, UL et al Factors Influencing the Emergence and Spread of HIV Drug Resistance Arising from Rollout of Antiretroviral Pre-Exposure Prophylaxis (PrEP). PLoS ONE 2011;6(4):e18165. | Population impact model not including costs assessment |
| Campbell JD, et al. Antiretroviral Prophylaxis for Sexual and Injection Drug Use Acquisition of HIV. Am J Prev Med. 2013 Jan;44(1 Suppl 2):S63-9. | Review |
| Daou, S et al HIV prevention using pre-exposure prophylaxis. Revue de Medicine Interne 2011;32(11):658-662. | Review |
| Granich R et al Expanding ART for treatment and prevention of HIV in South Africa: estimated cost and cost-effectiveness 2011-2050. PLoS ONE 2012 7(2):e30216. | PrEP not evaluated |
| Grant R et al Cost-effectiveness analysis of HIV chemoprophylaxis [Abstract THLB0102]. AIDS (2006). Toronto: IAS: http://library.iasociety.org/AbstractView.aspx?confID=2006&abstractID=51363. | Not enough information to review |
| Grant, RM. Antiretroviral Agents Used by HIV-Uninfected Persons for Prevention: Pre- and Postexposure Prophylaxis. Clinical Infectious Diseases 2010;50:S96-S101. | Opinion, review |
| Hankins CA, Dybul MR. The promise of pre-exposure prophylaxis with antiretroviral drugs to prevent HIV transmission: a review. Curr Opin HIV AIDS. 2013 Jan;8(1):50-8. | Review |
| Horberg M, Raymond B. Financial Policy Issues for HIV Pre-Exposure Prophylaxis: Cost and Access to Insurance. Am J Prev Med. 2013 Jan;44(1 Suppl 2):S125-8. | Review |
| Hurt CB et al. Pre-exposure prophylaxis and antiretroviral resistance: HIV prevention at a cost? Clin Infect Dis 2011 Dec;53(12):1265-70. | Review |
| Kelesidis, T et al. Preexposure prophylaxis for HIV prevention. Current HIV/AIDS reports 2011;8(2):94-103. | Review |
| Keller SB Smith D. The price of tenofovir-emtricitabine undermines the cost-effectiveness and advancement of pre-exposure prophylaxis. AIDS 2011 Nov 28;25(18):2308-10. | Correspondence, review |
| Kim, SC et al. Planning for pre-exposure prophylaxis to prevent HIV transmission: challenges and opportunities. Journal of the International AIDS Society2010;13;24. | Opinion, review |
| Lee, DH et al. Preexposure Chemoprophylaxis for HIV Prevention. NEJM 2011;364(14):1372-1373. | Correspondence, review |
| Leibowitz, AA et al. A US Policy Perspective on Oral Preexposure Prophylaxis for HIV. American Journal of Public Health 2011;101(6):982-985. | Policy perspective and review |
| Mayer, KH et al. Chemoprophylaxis for HIV Prevention: New Opportunities and New Questions. JAIDS 2010:55;S122-S127. | Review |
| Molina J. Pre-exposure prophylaxis: where are we in Europe? J Int AIDS Soc. 2012 Nov 11;15(6):18069. | Review |
| Okwundu CI et al. Antiretroviral pre-exposure prophylaxis (PrEP) for preventing HIV in high-risk individuals. Cochrane Database Syst Rev. 2012 Jul 11;7:CD007189. | Review of clinical trials, no costs assessment included |
| Prinja S et al. Cost effectiveness of targeted HIV prevention interventions for female sex workers in India. STI 2011; 87(4):354-361. | PrEP not evaluated |
| Schackman BR, Eggman AA. Cost-effectiveness of pre-exposure prophylaxis for HIV: a review. Curr Opin HIV AIDS. 2012 Nov;7(6):587-92. | Review |
| Schwartlander B et al. Towards an improved investment approach for an effective response to HIV/AIDS. Lancet 2011 377:2031-2041. | Review |
| van de Vijver, DAMC .The risk of HIV drug resistance following implementation of pre-exposure prophylaxis. Current Opinion in Infectious Diseases 2010;23(6):621-627. | Review |
| Vissers DC et al. The impact of pre-exposure prophylaxis (PrEP) on HIV epidemics in Africa and India: a simulation study. PLoS One 2008; 3: e2077. | Population impact model not including costs assessment |
| Weber, J et al. Postexposure prophylaxis, preexposure prophylaxis or universal test and treat: the strategic use of antiretroviral drugs to prevent HIV acquisition and transmission. AIDS 2010;24:S27-S39. | Review |
