# Supplementary material for: The Cost and Impact of Scaling Up Pre-exposure Prophylaxis for HIV Prevention: A Systematic Review of Cost-Effectiveness Modelling Studies
Source: PLoS Med. 2013 Mar 12;10(3):e1001401. doi: 10.1371/journal.pmed.1001401 (PMC3595225; doi:10.1371/journal.pmed.1001401)
Supplement: Table S2 — Summary of conclusions of articles included in the review. (DOCX) [file pmed.1001401.s002.docx]

**The cost and impact of scaling-up pre-exposure prophylaxis for HIV prevention: a systematic review of cost-effectiveness modelling studies.** Gomez GB, Borquez A, Case KK, Wheelock A, Vassall A, Hankins (**SUPPLEMENTARY MATERIAL - results)**

**Table S2:** Summary of conclusions of articles included in the review.

| Reference | Study conclusions |
| --- | --- |
| Generalised epidemics in southern Africa | |
| Abbas[1] | PrEP found to be cost effective. The main determinants of PrEP impact were: the effectiveness of PrEP, changes in sexual behaviour of the individuals on PrEP, adherent long term use and the level of coverage. Drug resistance did not emerge as a key determinant of impact. Targeting by sexual activity could have a significant epidemiological impact, while targeting by age group had less of an impact than a non-targeted approach. |
| Pretorius[2] | The model aimed to compare PrEP and universal test and treat (UTT). Non-targeted PrEP coverage doe not realistically have an effect comparable to the effect of UTT on incidence reduction. Targeted PrEP interventions can achieve a non-negligible reduction in incidence. However, PrEP cost-effectiveness and its impact on incidence would be considerably reduced should UTT be introduced in South Africa or should the rate of expansion of the current national ART program increases. |
| Hallett[3] | PrEP is a cost-effective intervention in sero-discordant couples. The key determinants of PrEP cost-effectiveness are PrEP effectiveness, relative costs of PrEP and ART delivery, and couples’ sexual behaviour. If risk reduction counselling achieves a change in couples risk behaviours, earlier initiation of ART becomes more cost-effective than PrEP. However, if PrEP effectiveness is high, then PrEP continues to be a cost-effective alternative. |
| Williams [4] | The use of the tenofovir gel to prevent HIV infection is a highly cost-effective intervention. A key determinant of the cost per infection averted is HIV incidence. If gel use can be targeted at women at highest risk, the impact of the gel would be increased. In particular, this is an opportunity for targeting young women In South Africa. However, the authors did not quantify this possibility (the model is not age-stratified). |
| Walensky[5] | Vaginal PrEP would be a highly cost-effective intervention among women in South Africa. Cost savings could be achieved should PrEP be prioritised to high risk populations (high incidence of HIV infection). Key determinants of PrEP cost-effectiveness, other than risk, are the effectiveness and PrEP drug costs. Improvements in adherence will maximise the effectiveness of a PrEP program. The authors found that the clinical benefits of PrEP at the population level overwhelmingly offset any plausible level of adverse reactions, resistant breakthrough infections, or changes in behaviour. |
| Alistar[6] | Expanded ART coverage, especially for individuals in early disease stages, is the most attractive strategy for HIV prevention. PrEP may be cost-saving, if it can be delivered to individuals at greater risk of infection. |
| Cremin[7] | Population-level maximal cost-effectiveness is achieved by providing ART to more infected individuals earlier rather than providing PrEP to uninfected individuals. A PrEP intervention is unlikely to generate a large reduction in HIV incidence. However, early ART alone cannot reduce HIV incidence to very low levels and PrEP can be used cost-effectively in addition to earlier ART to reduce incidence further. |
| Concentrated epidemics among MSM in high income countries | |
| Desai[8] | PrEP among high-risk MSM in large metropolitan areas could result in significant numbers of HIV infections prevented and be cost-effective under many scenarios. Key determinants of the results were efficacy, mechanism of protection, and coverage. For instance, an enrollment of 2.5% of high-risk MSM did not prevent enough HIV infections to justify the intervention. Assumptions about ART costs did not affect whether cost-effectiveness ratios fell within thresholds of interest. The authors found that even a small increase in sexual partners could offset te gains from a PrEP intervention. |
| Paltiel[9] | PrEP can substantially reduce the lifetime risk of HIV infection in the US but it remains an unattractive intervention, from a US-based cost-effectiveness perspective. Key determinants of PrEP cost-effectiveness are efficacy, prioritisation to higher risk groups (e.g. younger MSM), and pricing. Even though the authors adopted pessimistic base case set of assumptions with regards to resistance, their findings do not identify resistance in breakthrough infections to be a driver of PrEP cost-effectiveness. |
| Koppenhaver[10] | PrEP may have a significant impact on the HIV epidemic but at a high cost and was not considered a cost-effective intervention in a population of MSM in the USA. Two main drivers of the high cost-effectiveness ratios: PrEP effectiveness leading to more HIV-negative individuals receiving PrEP over time and therefore increases in PrEP costs; the incremental QALYs saved from PrEP became far greater near the end of the time horizon. |
| Juusola[11] | PrEP for HIV prevention in MSM could have an important impact on the HIV epidemic in the United States but affordability seems uncertain. Prioritising high-risk MSM would be essential to improve the cost-effectiveness estimates. A reduction of the cost of PrEP could also make PrEP a more attractive alternative in the general MSM population. |
| Concentrated epidemics among MSM in low and middle income countries | |
| Gomez[12] | The model shows an important epidemiological impact and cost-effectiveness of PrEP use, largely driven by PrEP conditional efficacy, coverage, prioritisation strategy, time to scale up, and risk compensation behaviour. The benefits will be greater if the intervention is prioritised to those most vulnerable to infection (MSM at highest risk). Risk compensation could affect the estimates. A higher coverage and rapid scale-up strengthens the impact on incidence. Despite cost-effectiveness at apparently feasible levels of coverage and uptake, PrEP requires considerable expenditures and human resources. |
| Concentrated epidemics among people who inject drugs in low and middle income countries | |
| Alistar[13] | Oral PrEP could be considered as part of a cost-effective intervention package for HIV control in IDU. However, focusing on MMT and ART access will give the biggest return for investments. Costs should decrease for PrEP alone may become cost-effective. |

REFERENCES

1. Abbas UL, Anderson RM, Mellors JW (2007) Potential impact of antiretroviral chemoprophylaxis on HIV-1 transmission in resource-limited settings. PLoS One 2: e875.

2. Pretorius C, Stover J, Bollinger L, Bacaer N, Williams B (2010) Evaluating the cost-effectiveness of pre-exposure prophylaxis (PrEP) and its impact on HIV-1 transmission in South Africa. PLoS One 5: e13646.

3. Hallett TB, Baeten JM, Heffron R, Barnabas R, de Bruyn G, et al. (2011) Optimal uses of antiretrovirals for prevention in HIV-1 serodiscordant heterosexual couples in South Africa: a modelling study. PLoS Med 8: e1001123.

4. Williams BG, Abdool Karim SS, Karim QA, Gouws E (2011) Epidemiological impact of tenofovir gel on the HIV epidemic in South Africa. J Acquir Immune Defic Syndr 58: 207-210.

5. Walensky RP, Park JE, Wood R, Freedberg KA, Scott CA, et al. (2012) The Cost-Effectiveness of Pre-Exposure Prophylaxis for HIV Infection in South African Women. Clin Infect Dis.

6. Alistar SS, Grant P, Bendavid E (2012) Paper #1081 - An Economic Analysis of ART and PrEP for HIV Prevention: South Africa (<http://www.retroconference.org/2012b/Abstracts/44724.htm)>. 19th Conference on Retroviruses and Opportunistic Infections. Seattle.

7. Cremin I, Alsallaq R, Dybul M, Piot P, Garnett G, et al. (2013) The new role of antiretrovirals in combination HIV prevention: a mathematical modelling analysis. Aids 27: 447-458.

8. Desai K, Sansom SL, Ackers ML, Stewart SR, Hall HI, et al. (2008) Modeling the impact of HIV chemoprophylaxis strategies among men who have sex with men in the United States: HIV infections prevented and cost-effectiveness. AIDS 22: 1829-1839.

9. Paltiel AD, Freedberg KA, Scott CA, Schackman BR, Losina E, et al. (2009) HIV preexposure prophylaxis in the United States: impact on lifetime infection risk, clinical outcomes, and cost-effectiveness. Clin Infect Dis 48: 806-815.

10. Koppenhaver RT, Sorensen SW, Farnham PG, Sansom SL (2011) The cost-effectiveness of pre-exposure prophylaxis in men who have sex with men in the United States: an epidemic model. J Acquir Immune Defic Syndr 58: e51-52.

11. Juusola JL, Brandeau ML, Owens DK, Bendavid E (2012) The Cost-Effectiveness of Preexposure Prophylaxis for HIV Prevention in the United States in Men Who Have Sex With Men. Ann Intern Med 156: 541-550.

12. Gomez GB, Borquez A, Caceres CF, Segura ER, Grant RM, et al. (2012) The potential impact of pre-exposure prophylaxis for HIV prevention among men who have sex with men in Lima, Peru PLoS Med 9: e1001323.

13. Alistar SS (2011) TR2-2: Effectiveness and cost-effectiveness of oral pre-exposure prophylaxis for injection drug users in mixed HIV epidemics (<http://smdm.confex.com/smdm/2011ch/webprogram/Paper6553.html)>. 33rd Annual Meeting of the Society for Medical Decision Making Hillsborough, USA.
