# Supplementary material for: The Cost and Impact of Scaling Up Pre-exposure Prophylaxis for HIV Prevention: A Systematic Review of Cost-Effectiveness Modelling Studies
Source: PLoS Med. 2013 Mar 12;10(3):e1001401. doi: 10.1371/journal.pmed.1001401 (PMC3595225; doi:10.1371/journal.pmed.1001401)
Supplement: Text S2 — Protocol for systematic review (version 2). (PDF) [file pmed.1001401.s004.pdf]

Protocol for systematic review (version 2)

Title: Potential cost-efectiveness of pre-exposure prophylaxis programmes for HIV prevention

Authors: Gomez GB[1], Borquez A[2], Case KK[2], Wheelock A[3], Vassall A[4], Hankins C[1,4]

[1] Department of Global Health, Academic Medical Centre, University of Amsterdam and Amsterdam Institute for Global Health and Development, The Netherlands
[2] School of Public Health, Imperial College London, UK
[3] Centre for Patient Safety and Service Quality, Imperial College London, UK
[4] London School of Hygiene and Tropical Medicine, UK

Started 1 January 2012
Revised 30 August 2012

Contact details
Gabriela B Gomez
Amsterdam Institute for Global Health and Development
Trinity Buildings, Building C
Pietersbergweg 17, PO Box 22700
1100 DE Amsterdam, The Netherlands

T +31 (0)20 566 1328
F +31 (0)20 566 9557
E g.gomez@aighd.org

1. Objectives

The overall aim of this review is to assess cost-effectiveness models that have evaluated the population impact of the scale-up of a potential PrEP intervention. The specific objectives are:

- to describe modelling approaches of cost-effectiveness analyses of PrEP;
- to compare the effects of modeller's assumptions on the presented results;
- to explore the potential impact of five issues associated with PrEP implementation across epidemiological and geographical contexts: prioritisation strategies in the design of the programme, adherence, behaviour change due to PrEP use, toxicity of PrEP drugs, and influence of a PrEP introduction on circulating resistance.

2. Criteria for inclusion of studies in the review

Studies included in the review must adhere to the following criteria

Inclusion Criteria - method: The review focuses on estimated health impacts and costs of a potential PrEP intervention. Rather than reviewing empirical studies, we will aim to review studies of estimations and projections of future impact. Therefore, we will review only modelling studies reporting both costs to the health service and benefits to the population of a potential scale up of a PrEP programme. There will be no restriction on the type of model designed, i.e. we will include models built with and without a transmission component.

Inclusion Criteria - setting: We are interested in reviewing the potential impact of PrEP in different settings. As such we will review studies independently of the epidemiological setting (e.g. mode of transmission modelled: homosexual, heterosexual, injections or stage of the epidemic: generalized or concentrated), and the geography (e.g. developing and developed countries).

Inclusion Criteria - population: no restriction criteria. The analysis will compare potential impact across populations modelled but special attention will be paid to distinguish between different population types in the analysis.

Inclusion Criteria - outcome reported: included studies must report both health gains (effectiveness) and costs. However, the metric chosen to report health gain (effectiveness) will not be an inclusion criterion. It could include (but not restricted to) for instance cost/infection averted, cost/quality-adjusted life year (QALY) gained, or cost/disability-adjusted life year (DALY) averted.

Inclusion Criteria - intervention: studies must evaluate a roll out of a pre-exposure prophylaxis programme. Pre-exposure prophylaxis will be defined as the use by HIV-negative people of anti-retroviral treatment to prevent acquisition of HIV. This can include both topical and systemic PrEP products available or hypothetical.

Inclusion Criteria - publication status: studies included should have been published either as a peer-reviewed paper or an abstract in a peer-reviewed conference.

3. Search Strategy and selection procedure

Studies will be identified both manually and electronically. Electronic databases such as PubMed/Medline, ISI Web of Knowledge (including Web of Science, Current Contents Connect, Derwent Innovations Index, CABI : CAB Abstracts, and Journal Citation Reports), Centre for

Reviews and Dissemination databases (including DARE - Database of Abstracts of Reviews of Effects, NHS EED - NHS Economic Evaluation Database, and HTA database - health technology assessments) will be targeted from the start date of this review and no language or publication date restrictions will be placed on these searches. The searches will be broad to ensure that as many studies as possible are assessed and will include the following basic terms:

(HIV or "human immunodeficiency virus")
AND
(tenofovir or "pre-exposure prophylaxis" or chemoprophylaxis or PrEP)
AND
(cost or cost-effectiveness or cost-utility or cost-benefit)

These searches will be modified as necessary according to database. The exact search terms will be recorded as the search strategy is refined. One reviewer will keep a log of all searches performed by database and the results. A second reviewer will run the searches separately and compare the results.

The list of studies selected for further consideration will be compared by the two reviewers after the initial screening of all abstracts and titles. The full text reports of all studies selected for further examination will be assessed independently for inclusion. The two reviewers will then compare the final list of identified publications and, in case of disagreement as to whether to include a study, a third reviewer will be asked to assess the publication in question. A record will be kept of all studies included and excluded with the reasons for exclusion.

Citations and bibliographies of key publications will also be reviewed for additional relevant articles. Experts will be consulted for information of additional ongoing or published studies as well as theme leaders at UNAIDS and WHO. Websites of relevant organisations (e.g. International AIDS Economic Network) will also be performed. We refer to these search strategies as "manual searches".

4. **Data Management:**

Once the studies have been identified and selected for inclusion in the review, two reviewers will extract data independently onto a prepared excel spreadsheet and will be able to summarise the most important issues and results of each study in a "comments" field. Critical appraisal of the quality of included studies will be part of the data extracted (e.g. assumptions and structure of models developed) and will be included in the database. The resulting databases will be compared and, in case of differences, both reviewers will discuss the discrepancies. If the reviewers cannot arrive to an agreement, they will consult with a third reviewer.

5. Analysis.

We expect important heterogeneity in the studies selected. For the comparisons, we will describe and categorise studies based on:

The model structure:

- type of model – does the model includes a transmission component?
- epidemiological and geographical setting – concentrated/generalised epidemic, country of interest
- mode of transmission modelled – homosexual, heterosexual, injection, mother to child
- population of interest – gender, age, other relevant characteristic

- is there risk heterogeneity included in terms of different subgroups of the population of interest being at more or less risk that others?
- background HIV incidence or prevalence

PrEP interventions and implementation scenarios:

- PrEP efficacy or effectiveness assumptions,
- adherence to PrEP programme,
- individual adherence to PrEP,
- coverage and prioritisation strategies modelled: special attention to defining in detail the prioritisation scenarios. Prioritisation will be defined as PrEP being directed or offered to specific populations identified by sexual inclination, activity, age or any other characteristic.
- Is there any behavioural change expected after introduction of PrEP and described?
- resistance patterns and assumptions included in the model
- are toxicity effects due to PrEP use included in the model? If so, what effects where modelled and what are the related assumptions

Economic aspects of the analysis will follow guidelines for economic evaluations[1]:

- view point
- Rationale and description of alternative programmes compared
- Primary outcome of the evaluation
- Methods for costing and estimates (i.e. expected cost of PrEP drugs and programme implementation, service and above service costs, downstream ART cost averted included in the cost-effectiveness calculation, time horizon for the evaluation and discounting rates included).

It is expected that a meta-analysis will not be possible due to the nature of the studies reviewed. Therefore we will aim to present a narrative synthesis of the results. We will use international thresholds of cost-effectiveness to be able to compare across studies. The lead author will produce the first draft that will then be commented on by all co-authors. The draft should be in the form of an article suitable for publication submission in a peer-reviewed journal.

6. Duration

The review is expected to take nine months to complete.

1. Drummond MF, Jefferson TO (1996) Guidelines for authors and peer reviewers of economic submissions to the BMJ. The BMJ Economic Evaluation Working Party. Bmj 313: 275-283.
